# Economic Costs and Benefits of Community-Based Lymphedema-Management Programs for Lymphatic Filariasis in India

**DOI:** 10.4269/ajtmh.19-0898

**Published:** 2020-07-08

**Authors:** Larry Sawers, Eileen Stillwaggon

**Affiliations:** 1 Department of Economics, American University, Washington, District of Columbia;; 2 Department of Pediatrics, Tropical Medicine Section, Baylor College of Medicine, Houston, Texas;; 3 Department of Economics, Gettysburg College, Gettysburg, Pennsylvania

## Abstract

Lymphatic filariasis (LF) is endemic in 72 countries; 15 million persons live with chronic filarial lymphedema. It can be a disabling condition, frequently painful, leading to reduced mobility, social exclusion, and depression. The Global Program to Eliminate Lymphatic Filariasis aims to stop new infections and care for affected persons, but morbidity management has been initiated in only 38 countries. We examine economic costs and benefits of alleviating chronic lymphedema and its effects through simple limb care. We use economic and epidemiological data from 12 Indian states in which 99% of Indians with filariasis reside. Using census data, we calculate the age distribution of filarial lymphedema and predict the burden of morbidity of infected persons. We estimate lifetime medical costs and lost earnings due to lymphedema and acute dermatolymphangioadenitis (ADLA) with and without community-based limb-care programs. Programs of community-based limb care in all Indian endemic areas would reduce costs of disability by 52%, saving a per person average of US$2,721, equivalent to 703 workdays. Per-person savings are 185 times the program’s per-person cost. Chronic lymphedema and ADLA impose a substantial physical and economic burden in filariasis-endemic areas. Low-cost programs for lymphedema management based on limb washing and topical medication are effective in reducing the number of ADLA episodes and stopping progression of disabling lymphedema. With reduced disability, people can work longer hours per day, more days per year, and in more strenuous, higher paying jobs, resulting in important economic benefits to themselves, their families, and their communities.

Author’s Dedication. Eileen Stillwaggon passed away not long after this paper was accepted for publication by the AJTMH. Eileen’s participation in this project exemplifies qualities that she brought to all of her scholarship. She applied her skills as an economist to show that curing or preventing disease could be far cheaper than failing to do so. From her first piece of published research to her last, she worked to undermine racial and gender discrimination. A recurrent theme in her research were efforts to expose how racism distorts medical research and thus health policy. Her passion to make the world a better place will be sorely missed.

## INTRODUCTION

Lymphatic filariasis (LF) afflicts an estimated 120 million people in 72 countries of Africa, Asia, Oceania, and the Americas and is one of the diseases targeted for elimination by the World Health Assembly (World Health Assembly Resolution 50.29, 1997).^1–4^ India is the country with the largest number of infected persons, accounting for 42% of the global endemic population.^2^ The Global Programme to Eliminate Lymphatic Filariasis (GPELF), a public–private partnership to assist in advocacy, resource mobilization, and program implementation, embodies 2 “pillars”: stopping new infections using mass drug administration (MDA) and managing morbidity and preventing disability for persons already infected.^5^ As of 2017, only five endemic countries had not yet started MDA.^2^

An estimated 40 million people live with the disabling effects of LF, including about 15 million persons with chronic filarial lymphedema, primarily of the legs, but also of the arms, breasts, and scrotum, and 25 million men with hydrocele.^1,4^ Asymptomatic LF-infected persons are at life-long risk of developing lymphedema or hydrocele.^4^ Ultrasonographic and histological evidence shows lymphatic vessel damage in infected children long before the age at which lymphedema typically becomes evident, generally around the age of 20 years.^6,7^ Programs of morbidity management and disability prevention (MMDP) among infected persons, the second pillar of the GPELF, had been initiated in only 38 of the 72 endemic countries by 2017.^2^

In a 2016 study in Khurda district, Odisha (formerly Orissa), India, we demonstrated that the societal economic benefits of MMDP for filarial lymphedema were 130 times the costs of such interventions.^8^ The present article uses a methodology similar to that used in the Odisha study to estimate the costs and benefits of MMDP for all of India.

## NATURE OF THE DISEASE

Various species of mosquitoes, depending on world region, transmit larval forms of *Wuchereria bancrofti*, *Brugia malayi*, and *Brugia timori*. Adult worms damage lymphatic vessels, causing lymphedema that tends to worsen with age.^6,7^ The progressive worsening of filarial lymphedema is accelerated by recurrent episodes of acute dermatolymphangioadenitis (ADLA), disabling fever and intense pain lasting several days caused by bacterial infections.^9,10^ These infections generally enter the lower limbs where the skin is damaged by wounds or interdigital lesions.^9,11^ Each episode of ADLA further damages the lymph system and contributes to progression of chronic lymphedema, the severity of which has been classified into seven stages by Dreyer and others.^9,11,12^ Worsening lymphedema in turn increases vulnerability to entry lesions that lead to ADLA.^13^

### Prevention of increasing disability.

Simple, low-cost methods can prevent recurrent ADLA episodes or reduce their frequency and, thus, slow or end lymphedema progression.^14–29^ These methods include washing the legs and feet with soap, clean water, and a small cloth—paying special attention to interdigital crevasses, skin folds, and entry lesions; drying the limbs with clean towels; and applying antifungal creams, antibiotic ointments, or antiseptics. Also helpful is elevating affected limbs, exercising to improve lymphatic and venous drainage, and wearing shoes^9^ (see the online Supplement for additional discussion; see also ref. 8 and its online Supplement). Several studies have also found that limb hygiene was associated with reduced leg volume and regression in lymphedema stage.^17,26,30^

### The economic cost of LF.

Numerous studies in India have described the economic cost imposed by ADLA and filarial lymphedema.^31–35^ Those costs fall into two major categories: out-of-pocket medical costs (medication, payments to healthcare providers, and travel costs of patient and helper) and lost productivity in both paid employment and unpaid household labor. Both lymphedema and ADLA may compel workers to work fewer days per year or fewer hours per day, and they may earn a lower wage because they cannot engage in strenuous labor. Chronic lymphedema at advanced stages can be completely disabling. At intermediate stages, it leads to partial disability with substantial productivity loss. Together, productivity losses due to disability and out-of-pocket medical costs are an extraordinary economic burden for some of the poorest people in India.

### Previous studies of benefits of community programs for MMDP for LF in India.

Most community-based lymphedema-management programs for filariasis reported in the literature have been in India. In Kerala, 6 months after 1-day health camps to teach leg washing and care of bacterial entry points, 96% of participants reported reduced symptoms (redness, swelling, odor, wounds, and fever) after following the hygiene regimen.^19^ Also in Kerala, improved foot care was found to be an effective treatment even for the control group who received neither diethylcarbamazine (DEC) and ivermectin nor DEC and penicillin in placebo-controlled trials.^21,22^ A follow-up study a year later found a 72.5% reduction in the frequency of ADLA without additional interventions, confirming the lasting efficacy of education efforts to reduce morbidity.^23^ In Tamil Nadu, in a trial of three drugs to prevent ADLA, even the control group, practicing only leg washing, had reduced incidence of ADLA in the treatment year and beyond.^20^ In an LF-endemic district in Kerala, patients with grade II and III lymphedema (of four grades) were trained in limb hygiene in LF camps. Three months later, 25% fewer patients reported episodes of ADLA. In a companion study in Karnataka, 73% fewer patients had ADLA episodes^25^ (see also ref. 27). The results of a community-based limb-care program in Khurda district, Odisha, are consonant with the results discussed earlier.^30,36^

### A community-based program in Khurda district, Odisha, India.

A house-to-house census of filarial lymphedema was conducted in 2005 in the rural and peri-urban areas of Khurda district in Odisha, India. Among the 1.8 million persons canvassed, the study identified all residents with lymphedema, recording age, gender, number of ADLA episodes in the previous year, and lymphedema stage.^37,38^ A census offers considerable advantages for assessing the regional burden of morbidity compared with clinic or hospital data that include only persons who present for care. The latter may be less likely to include people in lower stages of lymphedema and with few episodes of ADLA, or who for whatever reason do not seek medical assistance. The census of households in Khurda district in Odisha generated the largest data set available of people with lymphedema and ADLA by age and gender.

The census was followed by a community-based lymphedema-management program in 1,447 villages using community health workers to train LF patients in leg hygiene and use of topical antibiotic and antifungal treatments.^30,38^ In other villages, 370 patients were recruited into a prospective cohort study that found a statistically significant decrease in perceived disability after 2 years in the program, with greater improvements in patients with moderate or advanced lymphedema. Patients also reported losing 2.5 fewer work days per month after 1 year in the program.^30,36,39^ In another study of the 370 patients in the limb-care program, ADLA episodes decreased 34% over 24 months, and there was both a drop in the percentage of persons whose lymphedema progressed (worsened) and an increase in the percentage of those whose lymphedema regressed (improved).^30^

### The context of a national public health policy in India.

In the present study, we are measuring the costs and benefits of a nation-wide program of MMDP in India. It is essential to understand the size and diversity of India, as well as the nature of its federal system of government. India is the seventh largest country in area and the second most populous with 1.3 billion people and 22 official languages. India has 29 states and seven territories that range in population from less than a hundred thousand to more than 200 million. Most (99%) Indians with filarial lymphedema live in 12 states, and 94% reside in only eight of those states^40,41^ (see Table 1).

**Table 1:** Population, share of India’s filarial lymphedema burden, and number of villages by state

State	Population (2018)^61^	State’s share (%) of persons with filarial lymphedema (2013)^40^	Number of villages (2011)^62^
Bihar	119,461,013	28.0	44,938
Andhra Pradesh	52,883,163	20.5	28,237
Uttar Pradesh	228,959,599	13.5	107,106
Odisha	45,429,399	10.3	51,527
West Bengal	97,694,960	10.3	40,997
Maharashtra	120,837,347	6.3	43,943
Tamil Nadu	76,481,545	5.2	16,369
Kerala	35,330,888	2.3	1,495
Karnataka	66,165,886	2.2	29,536
Gujarat	63,907,200	0.6	18,512
Madhya Pradesh	82,342,793	0.5	55,101
Assam	34,586,234	0.2	26,550

The government of India has attempted to promote national programs for specific diseases and conditions, but the resources for health and the priorities for health are unevenly distributed across the country. Ultimately, MMDP needs to be carried out at the level of the village, of which there are hundreds of thousands in endemic areas. A program of that scale may sound daunting, but numerous other activities are already accomplished at the village level. Commercial distribution networks and political networks reach virtually everyone in the country. For example, there were 1.035 million polling places in the 2019 election (with no voter residing more than 2 km from a polling place).^42^ Coca-Cola is distributed in more than 2.6 million retail outlets across the country, which attests to the strength of India’s transportation grid and logistical capacity.^43^

## METHOD

The objective of this study was to assess, from a societal perspective, the economic effect of MMDP programs that change the age distribution of lymphedema and ADLA in the Indian population over time. Using data from the Khurda census, we establish the age distribution of lymphedema stage and annual frequency of ADLA episodes. The age distribution of morbidity found in the Khurda census is consonant with clinical evidence on the biological process of LF over time^9,13,44,45^ In our analysis, we assume that the cross-section of morbidity by age cohort found in the census is a good representation of the progression of symptoms that could be expected as people with LF age and experience ADLA episodes that worsen lymphedema, not just in Khurda but elsewhere in India. We assume that without MMDP (the no-treatment scenario), each cohort would replicate the experience of older cohorts so that average lymphedema stage and average annual number of ADLA episodes would increase with age, reproducing the same degree of morbidity among today’s young people as is now seen in older people. In India as a whole, the age profile of disability is unlikely to have changed appreciably since the original Khurda census, given that MMDP is only gradually being introduced in India.

In the treatment scenario, we assume, based on experience reported in numerous studies cited earlier, that the community limb-care program on average halts the progression of lymphedema as patients age and reduces the frequency of ADLA.^14–30^ Consequently, we assume that people in each age cohort remain in their baseline lymphedema stage throughout their working lives. Based on the results of the limb-care program in Khurda, we assume that the frequency of ADLA episodes for each age cohort will be 34% lower than in the no-treatment scenario.^30^

Using Microsoft Excel (Microsoft Corporation, Redmond, WA), we estimate the economic cost of morbidity and disability over the working lives of affected persons without lymphedema management and the projected reduction in those costs that would result from implementation of a community-based lymphedema-management program in all endemic areas.

### Costs.

Using the two pairs of morbidity distributions—ADLAfrequency and lymphedema stage, with and without limb care—we calculate the economic cost for each scenario. The difference between them (the cost saving) is the economic benefit of lymphedema management. Costs are calculated from the societal perspective. We include out-of-pocket costs to patients for clinic visits, travel, and medications associated with ADLA episodes and lymphedema. Those costs are based on studies in India measuring per-episode cost of ADLA and annual spending on filarial lymphedema^31–35^ (the online Supplement provides sources and methods for estimating out-of-pocket costs).

We calculate lost productivity for patients due to chronic lymphedema and ADLA, which cause them to work fewer days per year, fewer hours per day, and/or with lower intensity of work. People living with filarial lymphedema are disproportionately poor and reside in rural areas, so our measure of lost productivity is for low-skilled agricultural workers, a conservative measure because it ignores higher paid workers with filarial lymphedema. In rural parts of a country such as India, income typically comes to the family in a variety of ways. In many families, only a small share of income takes the form of cash wages generated from either occasional or regular employment. In many households, an important share of household earnings is income in kind that consists of food, fiber, or fodder grown by men and women on land they own or rent. Another form of income is surplus agricultural production sold for cash or bartered. Women are key actors in the rural economy of India. Many work for cash wages, but the large majority perform domestic duties critical to the economic survival of the household economy.

There is little published information about productivity loss in different stages of lymphedema. Stage 7 is characterized by inability to perform activities of daily living such as walking, bathing, and cooking, which almost certainly precludes productive employment.^12^ In stages 1 and 2, lymphedema produces only a slight swelling of the lower limbs and thus is, likely, to produce little or no productivity loss. Estimating average productivity loss in stages 3–6 is more difficult. Based on the symptoms of stages 3, 4, and 5‒6 depicted in WHO publications, we estimate average productivity loss of 20%, 50%, and 75%, respectively. The results of our benefit–cost analysis are nearly unchanged if we assume 50% or 100% disability in stages 5 and 6 instead of 75%. Most of those who have lower limb lymphedema are in the lower stages where our productivity loss assumptions are zero or very low. A more precise estimate of productivity loss from lymphedema would not change our conclusions.

To estimate the value of a day’s labor (whether in cash or kind, whether inside or outside the home, for both women and men) in rural India, we use the average daily wage from *Wage Rates in Rural India* published by the Indian Ministry of Labour and Employment.^46^ These data report average wages on a monthly basis for men and for women over the crop year July 2017–June 2018 for six representative low-skilled agricultural occupations in the 12 Indian states in which 99% of Indian people with lymphedema live. (For a detailed discussion of calculation of wage rates, see the online Supplement.) We find the average wage for the 12 Indian states weighted by each state’s share of the country’s population living with filarial lymphedema and then find the average of men’s and women’s wages. That figure was 246 Indian rupees, equivalent to US$3.87 at the middle of the 2017–2018 crop year. All costs are estimated in U.S. dollars in January 2018.

To determine what economists call the present discounted value, costs and benefits are assumed to be worth less in the future than in the present.^47^ We discount future costs and benefits at 3% annually, the conventional discount rate in health economics. Real earnings (adjusted for inflation) are assumed to increase by 2.7% per year over the coming decades and real expenditure on medical care is projected to exceed increases in the consumer price index by 3% annually. Lost productivity is estimated over the working lives of all persons aged 18–72 years, and lifetime real out-of-pocket expenditure on medical care for filarial lymphedema and ADLA is estimated for those aged 8–72 years. Table 2 lists the parameter values used in the calculation of lifetime out-of-pocket medical costs and earnings loss (see the online Supplement for details).

**Table 2:** Parameter values: medical costs and earnings loss due to filarial lymphedema and ADLA

Parameter	Baseline estimate in January 2018*	Sources
Annual per-person out-of-pocket medical costs for chronic filarial lymphedema	US$10.09	32,33
Per-episode out-of-pocket medical costs for ADLA	US$1.23	31,34,35
Average duration of ADLA (lost work days)	4 days	34,63–70
Annual increase in real cost of medical care for chronic lymphedema and ADLA	3%	71–76
Annual discount rate	3%	47
Average daily wage rate	US$3.87	40,41,46
Annual increase in real wages	2.7%	41,71–84
Average number of days worked per year	260	
Percentage of work days lost annually because of chronic lymphedema		32,33,49,50,69,85
Stages 1–2	0%	
Stage 3	20%	
Stage 4	50%	
Stages 5–6	75%	
Stage 7	100%	

ADLA = acute dermatolymphangioadenitis.

* Derivation of values is explained in the Supplement.

We use conservative estimates for reduction in ADLA episodes and regression of lymphedema stage resulting from MMDP. Predictions for real (i.e., adjusted for inflation) wage growth over the next 60 years are subject to considerable uncertainty. Thus, we perform sensitivity analysis using a lower estimate of the rate of growth of real wages.

We use the cost of the intervention in the Khurda programs updated to 2018 U.S. dollar value, which is US$14.68. To find the average benefit per person of the intervention, we divide the total benefits by 15,853, the number of persons aged 8–72 years included in the analysis. We compare the benefit per person with the cost per person to calculate the benefit–cost ratio.

## RESULTS

**Figure 1.**
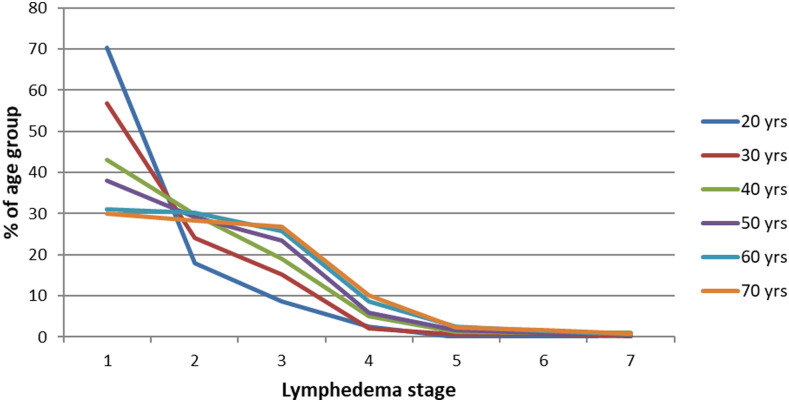
Age progression of lymphedema without morbidity management and disability prevention. This figure appears in color at www.ajtmh.org.

We identified 15,853 persons in the Khurda census with lower limb filarial lymphedema and derived the age distribution of morbidity due to lymphedema and ADLA episodes. Table 3 shows the distribution of lymphedema morbidity across age cohorts up to age 72 years. In the 20-year cohort (ages 18–22), most people (70.4%) are in Stage 1. Only 30% of those in the oldest cohort are in Stage 1. In Figure 1, each curve corresponds to the percentage of persons in each age cohort by lymphedema stage. The curves are increasingly convex when moving from younger to older cohorts, showing a higher percentage of people at higher stages of lymphedema. We also found that at higher stages, people experienced more episodes of ADLA on average. For example, only 6.4% of persons in stage 1 had three ADLA episodes in the previous year, whereas 15.1% of those in stage 7 had three ADLA episodes. Table 1 in the Supplement shows the full distribution of ADLA episodes by stage.

**Table 3:** Stage of lymphedema by age cohort in Khurda census, 2005

Age cohort (years)	Number of respondents	Percentage of age cohort at each stage of lymphedema								Average stage
		Stage of lymphedema								
		1	2	3	4	5	6	7	Total	
8–12	74	86.5	6.8	6.8	0.0	0.0	0.0	0.0	100.0	1.203
13–17	137	78.8	15.3	2.9	2.2	0.0	0.7	0.0	100.0	1.314
18–22	267	70.4	18.0	8.6	2.6	0.0	0.0	0.4	100.0	1.453
23–27	443	61.9	24.6	9.5	2.9	0.2	0.9	0.0	100.0	1.578
28–32	866	56.8	24.0	15.1	2.2	0.7	0.9	0.2	100.0	1.696
33–37	1,158	47.8	30.3	16.4	3.7	0.7	0.5	0.6	100.0	1.832
38–42	1,845	43.0	29.7	19.1	5.0	1.2	1.0	1.0	100.0	1.987
43–47	1,789	40.9	29.2	21.0	5.5	1.7	1.2	0.6	100.0	2.037
48–52	2,257	38.0	29.2	23.4	6.0	1.7	1.0	0.8	100.0	2.104
53–57	1,723	34.5	28.1	25.2	8.9	1.7	0.8	0.8	100.0	2.208
58–62	2,441	31.0	30.1	25.8	8.6	2.5	1.4	0.6	100.0	2.280
63–67	1,400	29.3	31.2	25.8	9.1	2.0	1.9	0.7	100.0	2.318
68–72	1,453	29.9	28.4	26.8	10.1	2.3	1.7	0.8	100.0	2.352
Total	15,853	39.5	28.6	21.9	6.6	1.6	1.1	0.7	100.0	2.084

*Source***:** Reproduced from Stillwaggon et al.^8^ Table 2.

### Economic cost without and with lymphedema management.

The present value of the lifetime benefit of lymphedema management (the reduction in economic cost) for this population averages US$2,721 per participant. That is equivalent to 703 days of work, based on the average daily wage (weighted by each state’s share of persons with filarial lymphedema) for low-skilled agricultural workers across India in 2018 of US$3.87.^48^ To implement and operate a community-based lymphedema-management program for 2 years would cost an estimated US$14.68 per patient.^30^ The average participant in the program can expect lifetime economic benefits that are 185 times the per-person cost of the program.

### Sensitivity test.

Most of the economic benefit of MMDP comes from fewer days of work lost (and thus higher productivity) due to reduced disability, not from lower out-of-pocket medical costs. Calculations of future economic gains produced by MMDP are thus predominantly dependent on the growth rate of wages. Assuming that the annual increase in the real average rural daily wage rate will be only 1% instead of our baseline assumption of 2.7% produces a 30% smaller increase in the per-person economic benefit of MMDP (from US$2,721 to US$1,911) and that in turn lowers the per-person benefit–cost ratio from 185 to 130. Our conclusion that MMDP for those with filarial lymphedema produces substantial economic benefits is thus robust to different assumptions about future real wage growth.

## DISCUSSION

This article presents the first attempt to calculate a benefit–cost ratio for all of India for an intervention aimed at mitigating the symptoms of filarial lymphedema. In 2000, Ramaiah et al.^49^ calculated the economic burden of filarial lymphedema for all of India calibrated with data from Tamil Nadu,^33,34,49,50^ but did not compare the burden with the cost of any intervention. Ours is the first attempt to find a benefit–cost ratio for MMDP interventions using a model parameterized with data from across India.

Although the present study of all India and an earlier one in Odisha used similar methodologies, the benefit–cost ratio found in this study (185) is substantially higher than the benefit–cost ratio found in the Odisha study (132).^8^ One reason for the difference is that Odisha has the lowest average wage among the 12 states in India with substantial prevalence of lymphedema, and the economic cost of lymphedema morbidity is mostly due to earnings loss. Furthermore, the Odisha study calculated wages and out-of-pocket costs of medical care centered on January 2009, whereas the calculations in this all-India study are centered on January 2018. The increase in real (adjusted for inflation) wages and medical costs in the 9 years between the two studies raises the benefit–cost ratio of MMDP substantially.

Lymphedema and episodes of ADLA in filariasis-endemic areas diminish the quality of life of affected persons because of pain, stigma, restricted mobility, and reduced participation in family and community life. They also impose a substantial economic cost on affected persons and their families and diminish the economic strength of communities. For these reasons, programs to provide care for persons with filarial lymphedema and ADLA (as well as hydrocele) in filariasis-endemic areas are mandated by the GPELF. (For a benefit–cost study of intervention to treat filarial hydrocele, see ref. 51.)

Beyond the ethical mandate to improve quality of life for affected persons, there are strong economic arguments for investing in the care of persons affected by filariasis, which the results of this research confirm. With appropriate limb care, patients are better able to support themselves and provide for their families. Children and other dependents of affected persons have improved nutrition and a greater opportunity to attend school when the wage earner is healthier. Family members are relieved of the burden of caring for persons who are bedridden because of ADLA or advanced lymphedema and can contribute better to household income and domestic tasks. The community’s economy is strengthened with fewer of its members disabled by lymphedema and ADLA and fewer of its families in poverty.

We find the average lifetime benefit of MMDP for those with filarial lymphedema is US$2,721. It is estimated that worldwide, there are close to 15 million persons with the condition. India’s share of the global burden is 42% or about 6.3 million (about a half percent of India’s population). If all of them received MMDP, the total benefit would be about US$17 billion in lifetime gains. In addition to people now living with the symptoms of filarial lymphedema in India, millions more who are now asymptomatic will likely become symptomatic in the coming years. The total lifetime benefits of providing training in limb-care to everyone with present or future symptoms of the disease are thus far more than US$17 billion. In comparison, the cost of MMDP for the 6.3 million Indians with filarial lymphedema is trivial, amounting to 0.003% of Indian GDP. The geography of the epidemic in India (most filarial lymphedema is found in just five states) may make it easier to mobilize a response, increasing its efficiency and concentrating its benefits.

### A public health approach: Integration with other interventions.

Programs that integrate morbidity management of filarial lymphedema with those for other diseases that require limb care, such as leprosy, diabetes, and podoconiosis, can be more effective and, with larger constituencies, better motivated. India has the world’s highest burden of Hansen’s disease (leprosy)—at least 60% of reported cases.^52–54^ Diabetes, now common in affluent countries, is a growing problem in low- and middle-income countries. In India, an estimated 8.7% of persons aged 20–70 years are diabetic, and thus, the disease is far more prevalent than filarial lymphedema.^55^ There are an estimated four million people globally with podoconiosis, for whom limb treatment is similar to that for LF.^56^ In India, podoconiosis is restricted to Manipur, Mizoram, and Rajasthan where the prevalence is 0.2%.^57^ The lessons learned from integrating limb-care programs can benefit Indian states and other countries, even if they have no LF.^57^ Integrated programs can help reduce the social isolation of disfiguring and debilitating diseases. Rehabilitating people in traditionally marginalized groups, which includes people with LF, leprosy, and podoconiosis, enables them to return to full participation in community life and carries an important message of inclusion.^58^

Programs to educate people in limb washing require access to clean water. Water, sanitation, and hygiene (WASH) programs are essential for limb care. They also can reduce breeding grounds for species of mosquito vectors of LF that flourish in open sewers. Reduced costs for limb-care programs, as well as reduced disability for LF patients, are important externalities that should be included in estimations of the benefits of WASH programs. Given the need for clean water, the recent drought in India poses special challenges for most of the states with the highest prevalence of filarial lymphedema (Karnataka, Maharashtra, Tamil Nadu, Andhra Pradesh, Uttar Pradesh, and Bihar).^55^

### Limitations.

We have noted numerous studies of MMDP for filarial lymphedema that have documented successful interventions and our modeling assumes that future MMDP will be as effective. Nevertheless, poorly implemented interventions may lead to little or no reduction in ADLA or improvement in lymphedema stage. A study in Pondicherry^59^ found that many clinicians and health workers were poorly informed about the difference between recommended techniques of limb care for filarial lymphedema and simply washing one’s leg while bathing.^9^ Participants in the study who did practice recommended leg hygiene techniques reported improvement in their condition. The Pondicherry study underscores the necessity of proper training of healthcare workers.

To model the economic impact over the lifetimes of those with filarial lymphedema and ADLA, we have made a number of conservative assumptions about labor markets, wages, productivity impact of disability, length of working life, cost inflation in health care, and other parameters. Although the present analysis shows substantial economic gains from MMDP—US$2,721 per person enrolled, we think that underestimates the economic benefits of lymphedema management. Our baseline estimate of productivity loss due to chronic lymphedema and ADLA, for example, was below the range found in several other studies. We did not include lost work time for youths until they reached the age of 18 years or for people older than 72 years, although young and old people in poor rural areas typically contribute to the work of the household to the extent they are able. In addition, to set the daily wage rate in our modeling, we chose only low-wage occupations in rural areas (omitting semi-skilled trades with higher wages and jobs in urban areas). Another assumption that leads to underestimation of the benefits of MMDP for filarial lymphedema is that systematic limb hygiene maintains the age structure of lymphedema morbidity and ADLA. Nevertheless, recent studies find that MMDP leads to net regression of the morbidity caused by filarial lymphedema.^15–30,36^ This study underestimates the costs of LF morbidity and benefits of MMDP in other ways by not including economic costs that do not fall on persons with chronic lymphedema and ADLA. We exclude costs to others, including society as a whole or the government. Subsidized care in government-run clinics, for example, is ultimately financed by the taxpayer. Reducing disease progression and disability reduces subsidies for medical care, a benefit to taxpayers that is not included in our analysis.

We have not included other second-order costs of chronic filarial lymphedema and ADLA such as lost work time of family caregivers for those disabled from ADLA and lymphedema or the impact on child nutrition and schooling, which could affect the child’s future earnings. By omitting those externalities, our calculations substantially understate the reduction in the economic cost of lymphedema and ADLA that a lymphedema-management program would generate. (See the Supplement for a discussion of other ways that conservative assumptions were used in parameterizing the model.)

## CONCLUSION

This study found that the lifetime present value of the benefit of community-based lymphedema management (the reduction in economic cost) was US$2,721 per participant. To implement and operate a community-based lymphedema-management program for 2 years would cost an estimated US$14.68 per patient.^30^ The average participant in the program can expect lifetime economic benefits that are 185 times the per-person cost of the program.

India has been one the fastest growing economies in the world since 2007, but that success can mask economic weakness in rural areas where the debilitating diseases of poverty are rife. The Indian government’s expenditure on health as a share of GDP is one-eighth the world average.^60^ MMDP programs are mandated as the second pillar of the GPELF. Low-cost interventions have been shown to be effective in reducing the frequency of episodes of ADLA and slowing progression of lymphedema. This study demonstrates that such interventions could produce very significant benefits to people and communities affected by filarial lymphedema, benefits that far exceed their costs.

## Supplemental file

Supplemental materials
