# Supplementary material for: Economic Costs and Benefits of Community-Based Lymphedema-Management Programs for Lymphatic Filariasis in India
Source: Am J Trop Med Hyg. 2020 Jul 8;103(1):295–302. doi: 10.4269/ajtmh.19-0898 (PMC7356420; doi:10.4269/ajtmh.19-0898)
Supplement: Supplementary file 1 [file tpmd190898.SD1.pdf]

Supplement S1: Detailed methods and sources used in "Economic costs and benefits of community-based lymphedema-management programs for lymphatic filariasis in India"

Larry Sawers,* Eileen Stillwaggon

*Corresponding author: Larry Sawers, lsawers@american.edu

This article uses economic and epidemiological data to estimate the economic impact of filarial lymphedema and its effects on out-of-pocket medical costs and worker productivity in areas of India with endemic lymphatic filariasis (LF). By reducing those medical costs and productivity losses, morbidity management programs produce economic gains for those who are helped and for their communities. This supplement provides detailed information on the sources used to determine parameter values and the method of calculating the results for our modeling of the economic costs of lymphedema and episodes of acute dermatolymphangioadenitis (ADLA) in a region with endemic LF. This study uses some data from and a method similar to that used in Stillwaggon *et al.*, "Economic costs and benefits of a community-based lymphedema-management program for lymphatic filariasis in Odisha State, India," *American Journal of Tropical Medicine and Hygiene*, 95(4), 2016.[1] Sections of this Supplement that explain methods and sources used in both articles repeat some of the material in the earlier online Supplemental Information, Detailed Methods and Sources.

1. *Efficacy of lymphedema management*: Numerous studies have provided evidence of the efficacy of simple programs of limb care in stopping or reversing progression of filarial lymphedema and reducing the number of episodes of ADLA. A recent systematic review and meta-analysis concluded that the "available evidence strongly supports the effectiveness of hygiene-based lymphedema management in LF-endemic areas" for the prevention of ADLA.[2] The meta-analysis found that participation in such programs was associated with decreased percentage of patients reporting at least one episode of ADLA (OR 0.29, 95% CI 0.12–0.47) and lower incidence of ADLA (OR 0.32, 95% CI 0.25–0.40).[2] Many successful lymphedema-management programs have been community-based educational campaigns to teach patients proper limb-care technique. Programs in Egypt, Nigeria, Burkina Faso, and Sri Lanka have produced substantial reductions in ADLA frequency.[3, 4, 5, 6]

Some studies suggest that MDA (mass drug administration) can reduce lymphedema and frequency of ADLA in persons already infected,[7, 8, 9, 10, 11, 12, 13, 14, 15, 16] but other studies do not.[17, 18, 19, 20] Consequently, available evidence does not warrant incorporating in the model any effect of MDA on lymphedema and ADLA.

2. A house-to-house census searching for persons with filarial lymphedema was conducted in the rural and peri-urban areas of Khurda District in Odisha (formerly Orissa), India, in 2005. The census identified all residents with lymphedema, recording age, gender, number of ADLA episodes in the previous year, and lymphedema stage.[21, 22, 23] The data collected in the census allow construction of the age profile of LF morbidity. We calculate out-of-pocket medical costs for lymphedema and ADLA for respondents who are 8 to 72 years old. We calculate lost productivity from ADLA and chronic disability of lymphedema for respondents 18 to 72 years old. Respondents older than 72 were omitted from the analysis of lost productivity because of small numbers and assumed low rates of labor force participation. Indian life expectancy is 66 years (http://data.worldbank.org/indicator/SP.DYN.LE00.IN). Respondents aged 8 to 18 were also not included in lost earnings calculation. Although many children do work, it is not

legal until age 14 and hazardous work is not legal until age 18. In any case, we base our estimates of productivity loss on *Wage Rates in Rural India*, which reports only fragmentary data for children.[24] We calculated economic cost of filarial lymphedema and ADLA for the 15,853 respondents in the census aged 8 to 72. A disproportionate share of respondents reported their age ending in 0 or 5. Hence, we organized the data in cohorts of five years, the youngest of which was 8 to 12 years and the oldest of which was 68 to 72 years.

The Khurda census is the only dataset that provides a detailed age profile of lymphedema morbidity. Our calculations assume that other states in India have an age structure of lymphedema morbidity similar to what was found in Odisha. In other words, we assume the age structure of LF morbidity in Odisha represents the medical characteristics of the disease in India, not anything specific to Odisha.

3. This study measures two categories of costs borne by those with filarial lymphedema. ADLA and the chronic symptoms of lymphedema give rise to out-of-pocket medical costs (medications, payments to health care providers, and transportation to clinics). The larger share of the cost of filarial lymphedema is the productivity loss imposed by lymphedema morbidity. The productivity loss comes in two ways. First, acute episodes (ADLA) during which patients are unable to engage in productive activities are temporarily disabling. Second, lymphedema can be chronically disabling such that patients are compelled to work fewer days per week and/or fewer hours per day. Their disability may force a reduction in the intensity of their effort and may lead to a reduced pay per hour or per day.

4. We discuss 4 aspects of medical cost estimation: out-of-pocket per episode costs of medical treatment for ADLA, annual out-of-pocket costs of medical treatment for lymphedema patients, and medical price increases before and after 2018.

4a. *Out-of-pocket costs of medical treatment for ADLA per episode*: We calibrate our model with 3 studies in India that report per episode rupee cost of medical care for ADLA. We convert rupee cost at the time of the study into US dollars and then adjust for US dollar inflation between the time of the study and January 2018 (the midpoint of the crop year on which our average agricultural wage is measured). We use the authors' exchange rate where possible. Otherwise, we use exchange rates from the XE currency tables (www.xe.com/currencytables). This method underestimates, perhaps substantially, the true cost of ADLA episodes because it does not adjust for medical cost inflation between the time of the study and 2018 (discussed below in section 4c).

We found 3 studies with appropriate data. A study in Khurda District, Odisha in 2000–2001 by Babu and Nayak (2003, Table 1)[25] reports that ADLA patients paid an arithmetic mean of 61 rupees per episode of ADLA, or US$1.85 in 2018 US dollars. A study in the urban region of Pondicherry in south India by Nanda and Krishnamoorthy (2003, page 57)[26] reports mean per-episode treatment costs of 22 rupees or US$0.68 in 2018 US dollars. A study in rural Tamil Nadu by Ramaiah *et al.* (1998, Table 2)[27] reports information from which one can calculate the arithmetic mean of per-episode spending on ADLA of 21 rupees in 1994 or US$1.15 in 2018 US dollars. The simple mean of these three estimates of per-episode out-of-pocket medical expense is US$1.23 in 2018 US dollars. All 3 of these estimates of per-episode cost of ADLA are based on data for lymphedema and hydrocele patients combined. We have

found no evidence that episodes of ADLA for lymphedema and hydrocele patients produce different productivity losses or medical expenses.

We do not use the results of the study by Krishnamoorthy[28] since it only reports geometric means of treatment costs. The geometric mean of a highly skewed distribution is much lower than the arithmetic mean, which is the appropriate measure for our modelling. We also do not use Ramaiah *et al*.'s 2000 study of all India[29] since the results it presents are based on their 1998 study,[27] which are already incorporated into our calculations.

4b. *Annual out-of-pocket costs of medical treatment for lymphedema patients*: We calibrate our model with studies in India that report annual rupee cost of medical care for filarial lymphedema. We convert the rupee cost at the time of study to US dollars in 2018 using the same protocol as we do for ADLA medical expense described above. We found 2 studies with appropriate data. A study in Khurda District, Odisha, by Babu *et al*. (2002, page 34)[30] finds that the average male lymphedema patient spent 576 rupees annually and the average female spent 425 rupees, the weighted average of which was 478 rupees, which was US$15.43 in 2018 US dollars. (Those reported averages are geometric means, which are lower than arithmetic means. This estimate thus understates the cost of treatment and generates a more conservative estimate of the benefits of the intervention.) A study in Tamil Nadu by Ramaiah *et al*.(1999, page 22, Table 2)[31] finds that the average annual medical expense for chronic lymphedema patients was 98 rupees or US$4.75 in 2018 US dollars. The simple mean of the two estimates of annual medical expense for lymphedema is US$10.09 in 2018 US dollars.

We do not use the study by Nanda and Krishnamoorthy (2003, page 57).[26] They report per-visit treatment costs in south India, but do not report annual number of visits, so annual spending cannot be calculated. We also do not use Ramaiah *et al*.'s 2000 study of all India[29] since the results it presents are based on their 1999 study,[31] which are already incorporated in our estimated mean.

4c. *Medical price increases before 2018*: In the decade following the aforementioned studies of out-of-pocket medical expense for ADLA and lymphedema (2006-2015), real (after inflation) wages of agricultural laborers in India increased by 60 to 70%.[32, 33] That wage increase should have pushed up demand for medical care (demand for which is income-elastic), putting upward pressure on medical care prices. At the same time, rising wages in rural areas likely put upward pressure on the wages of medical-care workers in rural areas. That would also add to medical-services price increases since labor expense constitutes an important share of the cost of medical services. In addition, pharmaceutical prices in India have risen in recent years.[34] Thus, our measure of the cost of medical care likely underestimates by a substantial amount what filarial lymphedema patients actually spent on medical care in recent years. That, in turn, biases downward our measure of the benefits of MMDP. Although we do not adjust out-of-pocket medical expenses for inflation in Indian medical prices, we do increase the price of medical services for lymphedema patients at the same pace as US dollar inflation from the time the medical costs were measured until January 2018 since our model presents all values in US dollars.

4d. *Future annual real increase in out-of-pocket medical costs*: The forces driving up the real prices of medical services in recent years that were discussed in the previous paragraph will almost certainly continue into the future. Complicating the analysis is the sharp rise in healthcare costs in India at the end of 2018 that has continued into 2019. The increase is puzzling, according to the State Bank of India's Chief Economist Soumya Kani Ghosh, because it came at a time when rural incomes and

presumably rural demand for health care were collapsing.[35] Medical inflation in India is now expected to be 4 or 5 percentage points higher than the consumer price index (CPI) in the coming year.[36, 37, 38] Moreover, medical inflation is higher in rural areas than in urban areas.[34, 39] One possible explanation for the jump in medical inflation is the national health insurance scheme launched at the end of 2018 the aim of which is to insure 100 million Indian families for up to US$7100 annually.[34] Another factor producing medical inflation might be the weakening rupee that is pushing up the price of imported medicine. Pharmaceutical prices are the most important component of the medical inflation index in India.[34] The prices of domestically produced drugs, however, do not seem to be a driver of the current price surge in healthcare.[34, 39] It seems likely that domestically produced analgesics, antibiotics, and antifungals constitute the majority of self-care drug expenses for poor people. Lastly, some argue that unregulated private health care providers appear to be squeezing public providers out of the market and in the process putting upward pressure on the prices of medical services.[39]

Our measurement of the costs of treating filarial lymphedema requires assumptions about future increases in the real (adjusted for inflation) price of medical care for decades into the future. The recent one-year jump in medical inflation is insufficient evidence to make long-term predictions, especially when the sources of the current price surge are poorly understood. All of the sources cited in the previous paragraph are from the business news media and some of those cite professional economists, but the issue is not yet addressed in scholarly literature.

The rising cost of health care seems to be a global phenomenon. An international medical consulting firm (Willis Towers Watson) estimated medical inflation (in excess of increases in consumer prices in general) in 2016 and 2017 in 60 countries; it found zero or negative medical inflation in only 2 countries and 6.0% and 7.5% medical inflation in India in the two years.[37] We estimate the future annual growth rate in the real cost of health care in India to be 3%, which we think is a conservative estimate that could produce an underestimate of the economic benefits of MMDP.

5. Most of the economic benefit of MMDP comes from fewer days of work lost (and thus higher productivity) due to reduced disability, not from lower out-of-pocket medical costs. What follows describes how we measure earnings loss in the current year and into the future.

5a. *Average rural low-skilled daily wage:* Since those with filarial lymphedema in India tend to be poor people living in rural areas, we seek a measure of low-skilled rural earnings to measure life-time productivity loss. We estimate the average rural daily wage in representative low-skilled occupations during the crop year July 2017 through June 2018 across the 12 Indian states for which filarial lymphedema cases are reported (Andhra Pradesh, Assam, Bihar, Gujarat, Karnataka, Kerala, Madhya Pradesh, Maharashtra, Odisha, Tamil Nadu, Uttar Pradesh, and West Bengal).[40, 41, 42] We calculate an average wage for these 12 states weighted by each state's share of the national population with filarial lymphedema.[41, 42]

Wage Rates In Rural India[24] reports average rural daily wages for men and women for every month for 6 occupations (sowing, weeding, transplanting, harvesting, winnowing, and threshing). The average wage in those occupations has been described as "the representative wage for an agricultural labourer" in India.[33] For each of the 12 states, we take the unweighted (since the number of workers in each occupation is not given) mean daily wage for men and for women in those 6 occupations averaged over 12 months of the crop year to find the average rural low-skilled daily wage by state. Lastly, we take

the average of men's and women's wages. With these assumptions, we find the average daily wage in 12 states of India to have been US$3.87 in January 2018. We use the average daily wage thus calculated to measure productivity loss from lymphedema and ADLA for everyone regardless of how they are remunerated, whether they engage in productive activity such as food preparation or child care within the household, earn a cash wage outside the household, or produce food, fiber, or fodder on land owned or rented by the household, whether or not it was sold for cash or bartered or consumed within the household.

Using cash wage rates for agricultural workers as a proxy for the value of all productive activity conforms to the way economists approach the analysis of rural labor markets in developing countries. Men and women move in and out of the cash labor market on a daily or seasonal basis. In a sense, everyone is competing against everyone else in the market for labor, and that competition should enforce a rough parity on the value of everyone's productive activity.

Each state's share of India's filarial lymphedema cases was obtained from Srivastava *et.al.*[41] The same information is available from Indiastat,[42] based on a statement supplied to the lower house of the national parliament (the Lok Sabha) in answer to Unstarred Question (that is, one requiring a written answer) No. 2665 on September 12, 2011 and made available to the public by the national government's Press Information Bureau. Those data appear to understate substantially lymphedema prevalence in India. We suspect that the reported prevalence is so low because of the absence – except in Khurda – of household censuses of LF morbidity in India. The Khurda census found that only 10% of respondents were in stages 4–7. If reporting of lymphedema cases depends on attending clinics and if patients in lower stages of filarial lymphedema (and thus with less severe symptoms) are less likely to attend clinics, then patients in stages 1-3 are likely to be under reported.

We use each state's share of reported lymphedema cases to weight our estimate of the average wage for low-skilled agricultural work by state. If the undercount of low-stage lymphedema patients is similar across all states, that would not affect each state's share of lymphedema cases. Weighting agricultural wages using Srivastava *et al*.'s data is preferable to not weighting wages. Not surprisingly, weighted average wages are lower than unweighted average wages (since LF tends to be a disease of poverty and of poorer states). Not weighting wages by filarial lymphedema prevalence would raise our benefit/cost calculation by 14.5%, which only strengthens the case of MMDP.

5b. *Women's labor force participation*: In computing the average wage, we take the average of women's and men's wages. In rural India, women's participation in wage labor is one of the lowest in the world (and falling sharply in recent years),[43, 44] but their labor in the household economy in growing food, husbanding animals, bearing and rearing children, preparing meals, cleaning and mending clothes, and other tasks are profoundly important economic contributions that we treat as commensurate with cash wages.

5c. *A conservative measure of rural wages.* The loss in wages due to the disabilities imposed by filarial lymphedema is useful for estimating the economic impact of lymphedema on individuals and their families, as well as the impact of their reduced spending in the community. Because of employers' efforts to pay wages less than the value workers produce, wages can be a poor measure of a worker's contribution to output. Moreover, an employer who takes profits out of the community reduces spending

and thus income earned in local shops or markets. Similarly, merchants or brokers who buy goods grown by farmers do not pay farmers the full value of their output. The community loses if those brokers are not local residents. For all of these reasons, workers' earnings or farmers' crop sales are a conservative proxy for productivity.

Lymphatic filariasis is predominantly a disease of poverty and is predominantly rural. Nevertheless, a substantial share of those who suffer from LF are not poor and do not live in rural areas. On average, rural wages in India are lower than in towns and cities. Using wages of rural poor people thus produces a conservative measure of productivity loss imposed by lymphedema and ADLA.

5d. *Complexities in measuring rural earnings:* In most agriculture activities, there are large variations in the work load over the year. During periods of planting and harvesting, agricultural workers are typically very busy. At other times of the year, there is less work to do on land owned by the family and less wage work available. Our estimates of the daily wage averaged over the crop year and the annual days of work lost necessarily smooth over seasonal variation in agricultural production.

5e. *Effect of MMDP on local labor markets:* We assumed that workers who increase their participation in the paid labor force through improved management of their lymphedema would not push down average wage rates since those with LF morbidity make up a small fraction of the available labor force. Moreover, the expected increase in participation in the paid labor force will be spread over decades without posing a shock to labor markets. Any increase in earnings produced by improved lymphedema-management is likely to be spent in the local economy, thereby stimulating job growth and helping to offset downward pressure on wages from increased labor supply. Since we have not included a local multiplier effect in our analysis, the economic benefit of the intervention is substantially underestimated.

5f. *Annual increase in real daily wage*: We measure earnings loss attributed to filarial lymphedema from the present until the end of working life. Accordingly, we must account for how earnings loss will change over time. The 1980s and 1990s in India were decades of impressive real (adjusted for price inflation) wage growth for agricultural workers in India.[32, 45, 46, 47, 48] Between 1983 and 1999–2000, real rural wage growth for men and women averaged 3.3% annually (Table 2, page 13).[47] After the turn of the century, however, real wage growth in rural areas stagnated. Average real annual wage growth was –0.7% between 2000–2001 and 2007–2008. In contrast, between 2007 and 2016, real annual GDP growth in India exceeded 9% in 3 of those years and 7% in another 6 years.[49] Rural workers shared in the prosperity. Average real annual wage growth from 2008–2009 to 2015–2016 in rural areas was 9.8%,[32, 33, 48]

A lively debate among Indian labor economists has identified numerous factors that could account for the spurt in real wages. One likely source of real wage growth was the implementation of MGNREGA (Mahatma Gandhi National Rural Employment Guarantee Act) in 2006.[46, 47, 50, 51, 52] The act mandated that every rural family was eligible for up to 100 days annually of unskilled employment on public works projects. In the early years of the program, the average number of days worked grew rapidly and nominal wages were indexed to inflation in many states. The expansion of the program put upward pressure on wages in rural areas generally. Other factors that might help to explain the rise in agricultural wages in rural India between 2007 and 2013 were a boom in construction that siphoned off farm workers into building rural roads and urban structures, rural to urban migration, new patterns of rural-urban

linkages, a decline in female participation in paid employment, and a decline in the absolute number of workers in agriculture.[33]

Since 2014, however, real rural wages in India have again stagnated. In the four years between 2014 and 2018, real rural wages grew annually by only 0.87%.[48, 53, 54, 55] By 2017–2018, the unemployment rate in India was at its highest level since the early 1970s.[56] One explanation for the slowdown in wage growth is falling real wages in the MGNREGA program. Moreover, the number of rural workers in the MGNREGA program peaked in 2013 and then fell by two-thirds by 2016.[57] Since 2013, the global slowdown in economic growth, the collapse of international primary commodity prices, a major contraction in food prices, growing disruption in long-standing trading relationships among many countries and India, and a drought in India in 2015 and 2016 have contributed to the slowing of rural wage growth.[46, 58] In predicting Indian rural wages over the coming decades, one should note the recent tendency for real rural wages to deteriorate in some industrially advanced states in India, casting doubt on the ability of urban India's prosperity to raise rural laborers out of poverty.[32]

Future growth in rural wages in India is uncertain in the face of rapid economic change in the country and economic turmoil across the globe. Since 1993, real annual increases in wages in agriculture have averaged 2.7%.[47, 48, 53, 54] We set our baseline estimation of annual real wage growth at 2.7%, assuming that the experience of the past quarter century will continue in the coming decades. Nevertheless, the contours of real wage growth since 2000 suggest that the spurt in real wage growth between 2008 and 2014 may have been an anomaly. In the early 2000s and again since 2015, real annual rural wage growth in India averaged less than 1% and turned negative in some years.

Since most of the economic benefit of MMDP comes from fewer days of work lost (and thus higher earnings) due to reduced disability, not in lower out-of-pocket medical costs, calculations of higher future earnings produced by MMDP are heavily dependent on the assumed increase in average real earnings. We perform a sensitivity test by reducing our estimate of annual growth in the real wage in the coming decades from the 2.7% baseline to 1%.

5g. *Annual work days lost by patients with ADLA:* Annual work days lost from ADLA are determined by multiplying the annual frequency of ADLA as found in the Khurda census by 4.0 days per episode, which was the average ADLA duration reported in nine studies in India.[27, 28, 59, 60, 61, 62, 63, 64, 65]

5h. *Annual work days lost by patients with chronic lymphedema*: Lymphedema can reduce one's ability to engage in productive work, and we measure that as days of work lost annually. Several studies record reduced hours of labor per day, reduced physical output, absenteeism, and coping by performing less strenuous and lower paid jobs for people with lymphedema.[29, 30, 31, 63, 66, 67] Our estimate of the annual work days lost by patients with chronic lymphedema is a composite figure that represents all of the ways that lymphedema morbidity reduces productivity and earnings.

We estimate the days of work lost due to the disability imposed by filarial lymphedema for each patient by first estimating the number of days a fully occupied rural laborer without filarial lymphedema could expect to work in a year and then subtract the percentage of days lost at each stage of the disease. There is scant published information on the subject. In a recent study (February 2019), the National Sample Survey Office (NSSO) – a division of the Government of India's Ministry of Statistics and

Programme Implementation – found that workers "in villages worked 46–47 hours in a week during the July–June 2018 period." That survey was the first official estimate of working hours in rural India.[68] Those data indicate that the typical work week in rural India is about 6 days. Allowing for days off for religious festivals, national holidays, inclement weather, and illness, we estimate the typical work week to be 5 days. With that assumption, fully able laborers would work 260 days per year.

Although there are numerous estimates of lost productivity due to chronic filarial lymphedema, no study reports the degree of disability and earnings loss by stage of lymphedema, measured as hours lost, days of work lost (and thus lost wages), or reduced wage rates. The WHO defines lymphedema stages by disfigurement of the lower limbs with the exception of Stage 7, which is distinguished by the patient's inability to perform "activities of daily living."[69] For our baseline calculations, we assume that lymphedema patients in Stage 7 are unable to perform any productive work. We assume that lymphedema patients in Stages 1 and 2 do not lose any productivity due to symptoms of lymphedema and thus work 260 days per year. We assume that patients with Stage 3 lymphedema experience a 20% reduction in productivity, missing the equivalent of 52 days of work per year and patients with Stage 4 lymphedema experience a 50% reduction in productivity, equivalent to missing 130 work days per year. We assume that lymphedema patients in Stages 5 and 6 face a 75% reduction in productivity, foregoing the equivalent of 195 days of productive activity annually because of chronic lymphedema. The Khurda census allows us to compute the number of respondents in each age cohort at each stage of lymphedema. We multiply the number of respondents by the average daily wage and multiply that by the average annual work days lost in each cohort. We sum over all cohorts to determine the economic cost of chronic lymphedema from lost productivity.

Our assumptions about degree of disability at each stage of lymphedema produce an average of 28 lost work days annually for persons with chronic lymphedema in stages 1–7. In other words, those with the disability of filarial lymphedema were able to work on average 10.4% fewer days annually compared to those without disability. That 10.4% reduction in work time is substantially lower than other studies have found. Ramaiah *et al.* (1999, Table 4)[31] found that lymphedema patients worked 15.2% less than controls. Ramaiah *et al.* (2000*b*, Tables 3 and 4)[63] show that those with chronic lymphedema worked 13.7% less than controls in paid work and 13.0% less in unpaid domestic work. Babu *et al.* (2002, Table 3)[30] found males with lymphedema worked 15.4% less than controls and women worked 23.5% less. Babu *et al.* (2006, page 714)[66] measured a 20.2% drop in earnings for weavers with lymphedema (8.0% from lower wages and 12.2% from fewer hours worked). Similarly, Ramu *et al.* (1996, page 670)[67] found that male weavers with lymphedema produced 27.4% less cloth than those with without lymphedema.

We suspect that one of the reasons our estimate of average work time loss from filarial lymphedema is lower than other estimates is that the Khurda census visited every household in the district. Medically trained personnel examined everyone identified with lower-limb lymphedema and determined the person's stage (including Stage 0, who were then excluded from the analysis). Lymphedema in Stage 1 is very difficult to detect in the morning as the swelling subsides when the patient reclines at night. Observers might not distinguish even Stage 2 filarial lymphedema from other conditions. Some studies of work loss due to lymphedema might have been designed to include only those with higher stage lymphedema. Ramaiah,[31] for example, reports selecting study participants "with obvious and overt chronic filarial manifestation." Patients in lymphedema stages 1 and 2, however, are unlikely to have "obvious and overt" symptoms. Excluding them from the analysis increases the

measured work loss from lymphedema. Of the people selected for study in the Khurda census on which our calculations are based, 68% were in stage 1 or 2. Similarly, surveying patients who present to clinics will likely oversample higher stages. While persons in stages 1 and 2 at present may not lose work time, if untreated their condition can be expected to worsen.

6. *Discount rate*: We use an annual discount rate of 3%, which is the conventional rate used for analyzing health interventions.[70]

Table 1. ADLA episodes in previous year experienced by persons in each lymphedema stage in Khurda census, 2005

	Percentage of persons in each stage with ADLA episodes				
Stage of lymphedema	*0 ADLA episodes*	*1 ADLA episode*	*2 ADLA episodes*	*3 ADLA episodes*	Total
1	17.1	68.7	7.8	6.4	100.0
2	16.1	71.4	7.2	5.2	100.0
3	15.2	69.4	9.2	6.3	100.0
4	14.2	68.5	9.6	7.7	100.0
5	15.7	58.7	12.3	13.3	100.0
6	10.8	62.1	12.8	14.3	100.0
7	12.6	57.1	15.1	15.1	100.0
Average	16.1	69.3	8.2	6.4	100.0

Source: Reproduced from Stillwaggon *et al.* Table 3.[1]

REFERENCES

1. Stillwaggon E, Sawers L, Rout J, Addiss D, Fox L, 2016. Economic costs and benefits of a community-based lymphedema management program for lymphatic filariasis in Odisha State, India. Am J Trop Med Hyg 95: 877-884.
2. Stocks ME, Freeman MC, Addiss DG, 2015. The Effect of Hygiene-Based Lymphedema Management in Lymphatic Filariasis-Endemic Areas: A Systematic Review and Meta-analysis. PLoS Negl Trop Dis 9: 1-12.
3. El-Nahas H, El-Shazly A, Abulhassan M, Nabih N, Mousa N, 2011. Impact of basic lymphedema management and antifilarial treatment on acute dermatolymphangioadenitis episodes and filarial antigenaemia. J Glob Infect Dis 3: 227-232.
4. Akogun OB, Badaki JA, 2011. Management of adenolymphangitis and lymphoedema due to lymphatic filariasis in resource-limited North-eastern Nigeria. Acta Trop 120 Suppl 1: S69-75.
5. Jullien P, Somé JdA, Brantus P, Bougma RW, Bamba I, Kyelem D, 2011. Efficacy of home-based lymphoedema management in reducing acute attacks in subjects with lymphatic filariasis in Burkina Faso. Acta Trop 1205: 555-561.
6. Wijesinghe RS, Wickremasinghe AR, Ekanayake S, Perera MS, 2007. Efficacy of a limb-care regime in preventing acute adenolymphangitis in patients with lymphoedema caused by Bancroftian filariasis, in Colombo, Sri Lanka. Ann Trop Med Parasitol 101: 487-497.
7. Bockarie MJ, Tisch DJ, Kastens W, Alexander ND, Dimber Z, Bockarie F, Ibam E, Alpers MP, Kazura JW, 2002. Mass treatment to eliminate filariasis in Papua New Guinea. N Engl J Med 347: 1841-8.
8. Ramaiah KD, Ottesen EA, 2014. Progress and impact of 13 years of the Global Programme to Eliminate Lymphatic Filariasis on reducing the burden of filarial disease. PLoS Negl Trop Dis 8: e3319.
9. Mackenzie CD, Lazarus WM, Mwakitalu ME, Mwingira U, Malecela MN, 2009. Lymphatic filariasis: patients and the global elimination programme. Ann Trop Med Parasitol 103 Suppl 1: S41-51.
10. Meyrowitsch DW, Simonsen PE, Makunde WH, 1996. Mass DEC chemotherapy for control of bancroftian filariasis: comparative efficacy of four strategies two years after start of treatment. Trans R Soc Trop Med Hyg 90: 423-8.
11. Partono F, 1985. Treatment of elephantiasis in a community with Timorian filariasis. Trans R Soc Trop Med Hyg 79: 44-6.
12. Shenoy RK, Suma TK, Kumaraswami V, Rahmah N, Dhananjayan G, Padma S, 2009. Antifilarial drugs, in the doses employed in mass drug administrations by the Global Programme to Eliminate Lymphatic Filariasis, reverse lymphatic pathology in children with *Brugia malayi* infection. Ann Trop Med Parasitol 103: 235-47.
13. Tisch DJ, Alexander ND, Kiniboro B, Dagoro H, Siba PM, Bockarie MJ, Alpers MP, Kazura JW, 2011. Reduction in acute filariasis morbidity during a mass drug administration trial to eliminate lymphatic filariasis in Papua New Guinea. PLoS Negl Trop Dis 5: e1241.
14. Casley-Smith JR, Jamal S, Casley-Smith J, 1993. Reduction of filaritic lymphoedema and elephantiasis by 5,6 benzo-alpha-pyrone (coumarin), and the effects of diethylcarbamazine (DEC). Ann Trop Med Parasitol 87: 247-58.
15. Eddy BA, Blackstock AJ, Williamson JM, Addiss DG, Streit TG, Beau de Rochars VM, Fox LM, 2014. A longitudinal analysis of the effect of mass drug administration on acute inflammatory episodes and disease progression in lymphedema patients in Leogane, Haiti. Am J Trop Med Hyg 90: 80-8.
16. Malecela MN, Mwingira U, Mwakitalu ME, Kabali C, Michael E, Mackenzie CD, 2009. The sharp end—experiences from the Tanzanian programme for the elimination of lymphatic filariasis: notes from the end of the road. Ann Trop Med Parasitol 103 Suppl 1: S53-7.

17. Joseph A, Mony P, Prasad M, John S, Srikanth, Mathai D, 2004. The efficacies of affected-limb care with penicillin, diethylcarbamazine, the combination of both drugs or antibiotic ointment, in the prevention of acute adenolymphangitis during Bancroftian filariasis. Ann Trop Med Parasitol 98: 685-696.
18. Kerketta A, Babu B, Rath K, Jangid P, Nayak A, Kar S, 2005. A randomized clinical trial to compare the efficacy of three treatment regimens along with footcare in the morbidity management of filarial lymphoedema. Trop Med Int Health 10: 698-705.
19. Das L, Subramanyam Reddy G, Pani S, 2003. Some observations on the effect of Daflon (micronized purified flavonoid fraction of Rutaceae aurantiae) in bancroftian filarial lymphoedema. Filaria J 12: 5.
20. Dunyo S, Nkrumah F, Simonsen P, 2000. Single-dose treatment of *Wuchereria bancrofti* infections with ivermectin and albendazole alone or in combination: Evaluation of the potential for control at 12 months after treatment. Trans R Soc Trop Med Hyg 94: 437-443.
21. Rout J, Honorat EA, Williamson J, Rao G, Fox LM, 2008. Burden of Lymphedema due to Lymphatic Filariasis–Orissa State, India. Poster: American Society of Tropical Medicine and Hygiene Annual Meeting. New Orleans.
22. Fox LM, Rout J, Budge P, Prakash A, Aloke Michyari, Little KM, 2011. Quantifying the Economic Benefits of a Community-based Lymphedema Management Program - Orissa State, India. American Society of Tropical Medicine and Hygiene Annual Meeting. Philadelphia, PA.
23. Walsh V, Little K, Wiegand R, Rout J, Fox LM, 2016. Evaluating the burden of lymphedema due to lymphatic filariasis in 2005 in Khurda District, Odisha State, India. PLoS NTDs 10: e0004917.
24. Labour Bureau, 2019. Wage Rates in Rural India. Available at: http://labourbureaunew.gov.in/showdetail.aspx?pr_id=iBJEgR8%2bUFY%3d. Accessed Mar 15 2019.
25. Babu B, Nayak A, 2003. Treatment costs and work time loss due to episodic adenolymphangitis in lymphatic filariasis patients in rural communities of Orissa, India. Trop Med Int Health 8: 1102-1109.
26. Nanda B, Krishnamoorthy K, 2003. Treatment seeking behaviour and costs due to acute and chronic forms of lymphatic filariasis in urban areas in south India. Trop Med Int Health 8: 56-59.
27. Ramaiah KD, Ramu K, Guyatt H, Vijar Kumar KN, Pani SP, 1998. Direct and indirect costs of the acute form of lymphatic filariasis to households in rural areas of Tamil Nadu, south India. Trop Med Int Health 3: 108-115.
28. Krishnamoorthy K, 1999. Estimated costs of acute adenolymphangitis to patients with chronic manifestations of Bancroftian filariasis in India. Indian J Public Health 43: 58-63.
29. Ramaiah KD, Das PK, Michael E, Guyatt H, 2000. The economic burden of lymphatic filariasis in India. Parasitol Today 16: 251-3.
30. Babu B, Nayak A, Dahl K, Acharya A, Jangrid P, Mallick G, 2002. The economic loss due to treatment costs and work loss to individuals with chronic lymphatic filariasis in rural communities of Orissa, India. Acta Trop 82: 31-38.
31. Ramaiah K, Guyatt H, Ramu K, Vanamail P, Pani S, Das P, 1999. Treatment costs and loss of work time to individuals with chronic lymphatic filariasis in rural communities in south India. Trop Med Int Health 4: 19-25.
32. A. V. Jose, 2018. Agricultural wages in Indian states. Ind. J. Labour Econ 60: 333-345.
33. Himanshu, 2017. Growth, structural change and wages in India: recent trends. Ind. J. Labour Econ. 60: 309-331.
34. Dugal I, Nahata P, 2019. The mystery of India ’s higher health inflation. Bloomberg Quint. Mumbai: Bloomberg Quint.
35. World Health Organization (WHO) India, 2019. Diabetes. Available at: http://www.searo.who.int/india/topics/diabetes_mellitus/en/. Accessed Jul 23 2019.

36. Mercer Marsh, 2017. 2019 Global Medical Trend Rates Report. Available at: https://www.aon.com/getmedia/0c375f10-3b16-4d2d-a452-4ae86968525b/2019-Global-Medical-Trend-Report.pdf.aspx. Accessed July 16, 2019.
37. Willis Towers Watson, 2017. 2018 Global Medical Trends Survey Report. Available at: https://www.willistowerswatson.com/en-ID/insights/2018/11/2019-global-medical-trends-survey-report. Accessed July 16, 2019.
38. Dhasmana I, 2019. CPI inflation in health, education sectors in rural areas stumps economists. Business Standard. New Delhi: Business Standard.
39. Sinha S, Morecha M, 2019. High retail inflation in healthcare pinching pockets. The Hindu Business Line. Chennai, India: The Hindu Business Line.
40. Labour Bureau, 2019. Wage Rates in Rural India. Available at: http://labourbureaunew.gov.in/showdetail.aspx?pr_id=iBJEgR8%2bUFY%3d. Accessed Mar 15, 2019.
41. Srivastava PK, Bhattacharjee J, Dhariwal AC, Krishnamoorthy K, Dash AP, 2014. Elimination of lymphatic filariasis: current status and way ahead. J Commun Dis 46: 85-94.
42. Datanet India, 2019. Indiastat: Selected state-wise Number of Cases of Lymphoedema and Hydrocele Reported in India (2008 to 2013). Available at: https://www.indiastat.com/health-data/16/diseases/77/filaria/17809/stats.aspx. Accessed Mar 25 2019.
43. Kapurthala, 2018. Culture and the labour market keep India ’s women at home. The Economist. Haryana: The Economist.
44. World Bank, 2019. Labor force participation rate, female (% of female population ages 15+) (modeled ILO estimate). Available at: https://data.worldbank.org/indicator/SL.TLF.CACT.FE.ZS. Accessed Mar 20, 2019.
45. Chavan P, Bedamatta R, 2006. Trends in agricultural wages in India. Econ Political Wkly 41: 4041-4051.
46. Kundu S, 2018. Rural wage dynamics in India: What role does inflation play? RBI Working Paper Series, Department of Economic and Policy Research, Reserve Bank of India.
47. Himanshu H, Kundu S, 2017. Rural wages in India: recent trends and determinants. Indian Journal of Labour Economics 59: 1-28.
48. Das A, Usami Y, 2017. Wage Rates in Rural India, 1998–99 to 2016–17. Review of Agrarian Studies 7: 4-38.
49. OECD.STAT, 2017. Economic Outlook No 102 - November 2017. Available at: https://stats.oecd.org/index.aspx?queryid=51396. Accessed Mar 21 2018.
50. Basole Amit, 2017. Long-run trends in rural wages. liveMint. Dehli.
51. Reddy AA, 2013. Trends in rural wage rates: whether India reached Lewis turning point? SSRN Electronic Journal Sept.
52. Usami Y, 2012. Recent trends in wage rates in rural India: an update. Rev Agrarian Stud 2: 171-181.
53. Himanshu, 2019. India's farm crisis: decades old and with deep roots. The India Forum April 5.
54. Chakravarty M, 2018. Rural wage growth falters: Rural wage growth is now negative. LiveMint. Delhi: HT Media.
55. Goel V, 2019. From underwear to cars, India ’s economy is fraying. New Times. New York.
56. Jha S, 2019. Unemployment rate hit four-decade high of 6.1% in 2017-18: NSSO survey. Business Standard. New Delhi.
57. Sridhar GN, 2018. RBI paper points to sharp fall in rural wages since 2014. The Hindu BusinessLine. Chennai: the Hindu Group.
58. Anand N, 2018. Rural India left hurting as wages drop sharply under Modi government. Quartz India. New York: Quartz News.
59. Babu B, Nayak A, Dhal K, 2005. Epidemiology of episodic adenolymphangitis: A longitudinal prospective surveillance among a rural community endemic for bancroftian filariasis in coastal Orissa, India. BMC Public Health 5: 1-6.

60. Ramaiah K, Ramu K, Vijay Kumar K, Guyatt H, 1996. Epidemiology of acute filarial episodes caused by *Wuchereria bancrofti* infection in two rural villages in Tamil Nadu, south India. Trans R Soc Trop Med Hyg 90: 639-643.
61. Pani S, Yuvaraj J, Vanamail P, Dhanda V, Michael E, Grenfell B, Bundy D, 1995. Episodic adenolymphangitis and lymphoedema in patients with Bancroftian filariasis. Trans R Soc Trop Med Hyg 89: 72-74.
62. Abidha, Das LK, Yuvraj J, Vijayalaxmi G, Pani SP, 2008. The plight of chronic filarial lymphoedema patients in choice of health care and health care providers in Pondicherry, India. J Commun Dis 40: 101-109.
63. Ramaiah KD, Radhamani MP, John KR, Evans DB, Guyatt H, Joseph A, 2000. The impact of lymphatic filariasis on labour inputs in southern India: results of a multi-site study. Ann Trop Med Parasitol 94: 353-364.
64. Rao C, Chandrasekharan A, Cherian C, 1982. Frequency and duration of acute filarial attacks in persons in *Brugia malayi* endemic community. Indian J Med Res 75: 813-815.
65. Sabesan S, Krishnamoorthy K, Pani S, Panicker K, 1992. Mandays lost due to repeated attacks of lymphatic filariasis. Trends in Life Sciences 7: 5-7.
66. Babu B, Swain B, Rath K, 2006. Impact of chronic lymphatic filariasis on quantity and quality of productive work among weavers in an endemic village from India. Trop Med Int Health 11: 712-717.
67. Ramu K, Ramaiah KD, Guyatt H, Evans D, 1996. Impact of lymphatic filariasis on the productivity of male weavers in a south Indian village. Trans R Soc Trop Med Hyg 90: 669-70.
68. Staff reporter, 2019. India's working hours one of the longest among global peers: NSSO report. Moneycontrol News. Mumbai: Moneycontrol News.
69. Organization) WWH, 2001. Lymphoedema Staff manual: Treatment and prevention of problems associated with lymphatic filariasis. Geneva.
70. Corso PS, Haddix AC, 2003. Time Effects. Haddix AC, Teutsch SM, Corso PS, eds. Prevention Effectiveness: A Guide to Decision Analysis and Economic Evaluation. New York: Oxford University Press, 92-102.
